# Comparative Genome Analysis Provides Molecular Evidence for Reclassification of the Photosynthetic Bacterium *Rhodobacter sphaeroides* EBL0706 as a Strain of *Luteovulum azotoformans*

**DOI:** 10.3390/microorganisms9081754

**Published:** 2021-08-17

**Authors:** Haoyu Wang, Xiaoling Sha, Rui Li, Yijing Li, Himel Nahreen Khaleque, Yuxiu Zhang, Tsing Bohu, Zhihui Bai, Xuliang Zhuang

**Affiliations:** 1 School of Chemical and Environmental Engineering, China University of Mining & Technology (Beijing), Beijing 100083, China; bqt1800302025@student.cumtb.edu.cn; 2 Research Center for Eco-Environmental Sciences, Chinese Academy of Sciences, Beijing 100085, China; xiaolingshaxls@163.com (X.S.); ruili_st@rcees.ac.cn (R.L.); liyijing1221@hotmail.com (Y.L.); zhbai@rcees.ac.cn (Z.B.); xlzhuang@rcees.ac.cn (X.Z.); 3 University of Chinese Academy of Sciences, Beijing 100049, China; 4 Beijing Huachen Jiguang Technology, Co. Ltd., Beijing 101407, China; 5 CSIRO Land and Water, Private Bag No. 5, Wembley, WA 6913, Australia; himelnahreen.khaleque@csiro.au; 6 State Key Laboratory of Lunar and Planetary Sciences, Macau University of Science and Technology, Taipa 999078, Macao; 7 CSIRO Mineral Resources, Australian Resources and Research Centre, Kensington, WA 6151, Australia

**Keywords:** *Rhodobacter sphaeroides*, *Luteovulum azotoformans*, reclassification, genome comparison, phylogenetic

## Abstract

In this study, we conducted a genome-wide comparative analysis of a former *Rhodobacter sphaeroides* strain EBL0706, which is now recorded as *Luteovulum sphaeroides* EBL0706. The genome of EBL0706 was compared with that of *Luteovulum azotoformans* ATCC 17025, *Luteovulum azotoformans* KA25, and *Luteovulum sphaeroides* 2.4.1. The average nucleotide identity (ANI), tetra nucleotide signatures (Tetra), digital DNA–DNA hybridization (dDDH) values, comparative genome, and phylogenetic analysis proposed that EBL0706 is a strain of *Luteovulum azotoformans*. Functional annotations identified a total of 4034 protein-coding genes in the genome of EBL0706, including a complete photosynthetic gene cluster. This study provides genomic molecular verification for the strain EBL0706 to be reclassified to *Luteovulum azotoformans*.

## 1. Introduction

The *Rhodobacter* genus is comprised of heterogenous members showing flexibility in ecophysiology and metabolic capability [1,2,3]. Members of the genus can fix atmospheric nitrogen and carry out anoxygenic photosynthesis, thereby allowing them to adapt to various environments and play key roles in global biogeochemical cycles [4,5,6]. Furthermore, *Rhodobacter* species have been model organisms for studying bacterial photosynthesis [7]. Their single photosynthetic system consists of the light-harvesting complex I (LH1), the light-harvesting complex II (LH2), and the reaction center (RC) [8,9], showing structural and functional similarities to the light system II of higher plants [10].

The cells of the anaerobic culture of strain EBL0706 are ovoid and brown, with a diameter range from 0.8 μm to 1.2 μm. The cell can carry out binary fission reproduction. The cell has a single polar flagellum and a vesicular intima structure. The reddish aerobic culture of strain EBL0706 uses biotin as a growth factor. Small molecular organic matters, such as sodium acetate, sodium succinate, and glycerol, can support the growth of EBL0706. This is not the case, however, for sodium benzoate and sodium tartrate (Table S1) [11]. Previous studies have revealed the uniquity of “*Rhodobacter sphaeroides*” strain EBL0706 for its outstanding antioxidant capacity [12] and the ability to synthesize a variety of bioactive substances, such as carotenoids [13,14], chlorophylls [15], superoxide dismutase [16,17], and vitamin B12 [18]. In addition, studies have demonstrated the potential of this strain for environmental remediation on pollutants, such as dichlorvos [19] and oil [20].

Recently, the *Rhodobacter* genus was further reclassified as a new genus *Luteovulum* gen. nov. [6,21], and also had another genus name, *Cereibacter*, in the NCBI database. The genus *Luteovulum* awaits appropriate action by the research community to be transferred to another genus; we propose *Luteovulum* here as a temporary name noted in our study. In the NCBI database, the *Luteovulum* genus currently contains six species: *Luteovulum sphaeroides*, *Luteovulum johrii*, *Luteovulum ovatum*, *Luteovulum azotoformans*, *Luteovulum alkalitolerans,* and *Luteovulum* *changlensis.* Within the six species, 28 strains were identified (https://www.ncbi.nlm.nih.gov/taxonomy/?term=Luteovulum) (accessed on 10 January 2021). So far, the complete genome sequences of eight strains among them can be obtained from public databases. *Rhodobacter sphaeroides* strain EBL0706 is currently classified and deposited in the NCBI database as *Luteovulum sphaeroides* EBL0706.

Here, a combination of phylogenetic analyses, including comparative genomics, average nucleotide identity (ANI), tetra nucleotide signatures (Tetra), and digital DNA–DNA hybridization were used to reclarify the taxonomic position of “*Rhodobacter sphaeroides*” strain EBL0706 to *Luteovulum azotoformans*.

## 2. Materials and Methods

### 2.1. Medium and Growth Conditions

The EBL0706 culture was obtained from China General Microbiological Culture Collection Center (CGMCC) under the identity number of CGMCC No. 0645. The strain was inoculated and resuscitated in sterile Luria–Bertani (LB) medium (Solarbio, Beijing, China) (10.0 g/L tryptone, 5.0 g/L yeast extract, and 10.0 g/L NaCl; pH 7.0) at 32 °C for 24 h at 3000 lx light intensity.

### 2.2. Sequencing and Genome Assembly

The whole genome DNA was extracted by Bacterial Genomic DNA Extraction Kit (Solarbio, Beijing, China) according to the user manual. Then, the genome of strain EBL0706 was sequenced using Illumina HiSeq2000 (Illumina, San Francisco, CA, USA) and Pacific Biosciences II (Pacific Biosciences, San Francisco, CA, USA) sequencing platforms. High-quality reads were assembled by SOAPdenovo v2.04 [22]. Inner gaps that exist in the scaffolding were filled with GapCloser [23]. Pacific Biosciences SMART analysis software 1.2 was used to generate long “filtered sub-reads” from the instrument. The quality of the genome obtained was assessed through CheckM [24].

Data were analyzed on the Major BioCloud Platform (www.majorbio.com) (accessed on 11 December 2020). The complete genome project has been deposited in the National Center for Biotechnology Information (NCBI) as CP031750–CP031755.

### 2.3. Phylogenetic Tree

The target and reference 16S rRNA gene sequences were obtained from EzTaxon (https://www.ezbiocloud.net/) (accessed on 20 January 2021). MEGA 7.0 was used to construct 16S rRNA gene phylogenetic trees based on the neighbor-joining method. A rooted phylogenetic tree was constructed using RAxML (v. 8.2.8) software based on 20 single-copy core gene sequences, showing relationships between 10 whole genomes (Table S2) from the NCBI database. The RAxML analyses were run with rapid bootstrap analysis and 1000 replicates.

### 2.4. Sequence-Based Methods for Species Circumscription

According to the recommended cut-off values for species determination (<95% for ANIb and <0.989 for Tetra) [25,26], the calculation of average nucleotide identity based on BLAST (ANIb) and the correlation indexes of tetra nucleotide signatures (Tetra) were conducted using JspeciesWS (http://jspecies.ribohost.com/jspeciesws/#Analyse) (accessed on April 15 2021) [27,28]. The dDDH values were calculated using the Genome-to-Genome Distance Calculator (GGDC) web tool, (http://ggdc.dsmz.de/distcalc2.php) (accessed on April 15 2021), with Formula 2 [29] and a cut-off of 70% to determine the distance between the genomes [30].

### 2.5. Comparative Genomics

Comparative genomic analysis to verify homology was carried out using Sibelia Software [31]. Strains that phylogenetically close to EBL0706 in the 16S rRNA tree were selected for the comparative analysis. These strains were *L. azotoformans* ATCC 17025, *L. azotoformans* KA25, and *L. sphaeroides* 2.4.1 [32,33]. *L. azotoformans* ATCC 17025 is formerly *L. sphaeroides* ATCC 17025 in the NCBI database and *Rhodobacter azotoformans* ATCC 17025 in the American Type Culture Collection. Genomic sequences of these strains were obtained from the NCBI database.

## 3. Results and Discussion

### 3.1. Genome Assemblies and Features

The size of the complete genome of strain EBL0706 was determined as 4.438 Mbp, with an average GC content of 68.4% (Table 1).

**Figure 1:**
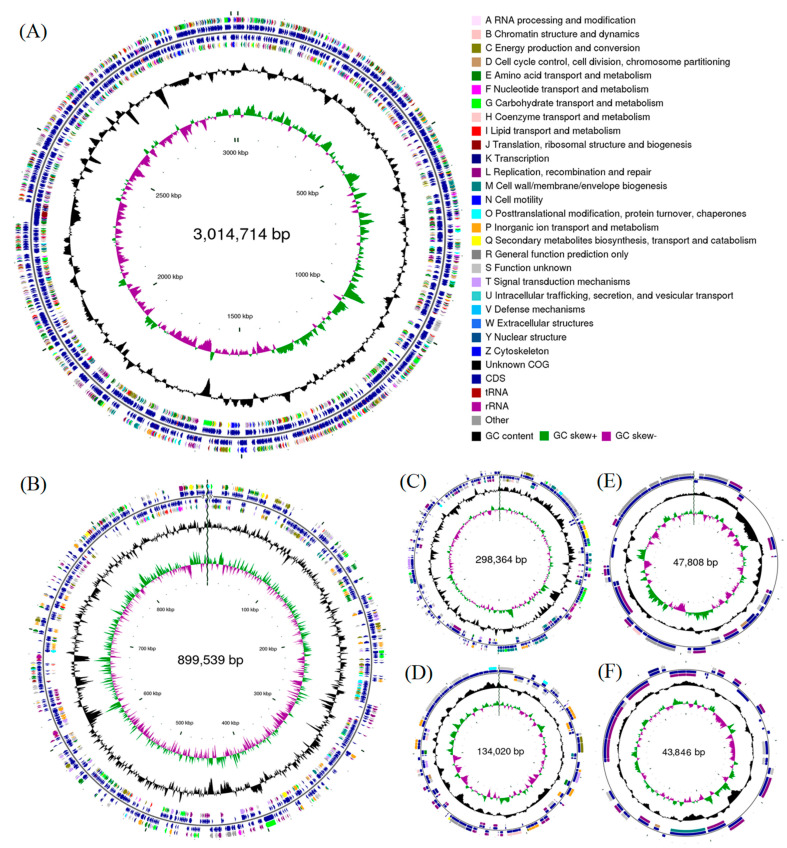
The complete genome map of strain EBL0706, including (**A**) chromosome 1, (**B**) chromosome 2, (**C**) plasmid A, (**D**) plasmid B, (**E**) plasmid C, and (**F**) plasmid D. The features of the marker are displayed from outside to inside as follows: coding sequences (CDSs), colored on clusters of orthologous groups (COG) functional categories, on the forward strand; tRNA and rRNA on the forward and reverse strand; CDSs on the reverse strand; GC content (plotted as the deviation from the average GC content of the entire sequence; outward plots as positive values and inward plots as negative values) and GC skew (G - /G + C, the leading chain and the lagging chain can be judged by the change of GC skew, generally the leading chain GC skew > 0, the lagging chain GC skew < 0).

The genome contains two chromosomes and four plasmids, including a 3,014,714 bp chromosome 1, an 899,539-bp chromosome 2, a 298,364-bp plasmid A, a 134,020-bp plasmid B, a 47,808-bp plasmid C, and a 43,846-bp plasmid D (Table S3). The mean G + C contents are 68.6%, 68.0%, 67.6%, 69.6%, 63.5%, and 64.7%, respectively (Figure 1). The complete genome contained 4034 protein-coding genes (Table S3), 12 rRNA operons coding 5S, 23S, and 16S rRNA, and 55 tRNA genes for 20 amino acids. These results were consistent with previous studies on *Rhodobacter* spp. [34,35,36].

Genome-wide analysis identified 19 genomic islands (GIs) in strain EBL0706. Genes related to these GIs are listed in Table S4. GIs of strain EBL0706 carry functional genes, such as ABC transporter protein family, heme biosynthesis protein *HemY*, integrases, and transposases [37].

Phagic genes, such as antibiotic resistance genes and virulence genes, can facilitate bacteria to adapt to hostile environments [38,39]. In the genome of strain EBL0706, five prophage elements and a total of 162 protein-coding genes were identified. Among the 162 proteins, 67 were phage proteins (Table S5).

### 3.2. Photosynthetic Genes

**Figure 2:**
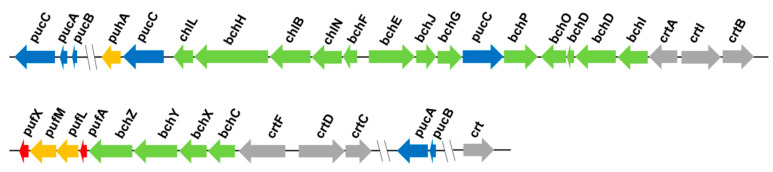
The photosynthetic gene cluster in strain EBL0706. Genes with different colors represent different functions (dark blue, *pucABC*, encoding LH2; orange, *puhA* and *pufML*, encoding RC; green, *bchCDEFGHIJXYZ* and *chlBLN*, encoding bacterial chlorophyll; red, *pufAX*, encoding LH1). The size and direction of arrows indicate the volume and direction of gene transcription. The characteristics of genes in Figure 2 were summarized in Table S6.

Strain EBL0706 harbors photosynthesis-relating operons, such as *puc*, *puf*, and *puh*, composed of the light-harvesting I (LH1), the light-harvesting II (LH2), and the reaction center (RC). The gene clusters encoding these photosynthetic apparatuses are shown in Figure 2. Operon *puc* encodes LH2. Operon *puf* encodes LH1, RC-L subunit, and RC-M subunit. Operon *puh* encodes RC-H subunit. In addition, *bch* and *crt* are involved in bacterial chlorophyll and carotenoid synthesis, respectively. The main pigments in the photosynthetic apparatus are bacterial chlorophyll and carotenoids. These pigments are bound to membrane proteins such as LH1, LH2, and RC to form a complete photosynthetic machinery (Figure 2). In strain EBL0706, the main function of *bchI* is photon absorption, while *crt* responds to damage from photo-oxidation, dissipates excess radiation energy, and maintains the photosynthetic apparatus. The light absorption of LH1 is affected by *pufX*, which can change the binding state of LH1 and RC to influence the electron transfer between the two functional assemblages. During photosynthesis, LH2 absorbs and transfers photons to RC through LH1, followed by charge separation. ATPs can be produced through the series of electron transfer [8].

### 3.3. Phylogenetic Analysis

**Figure 3:**
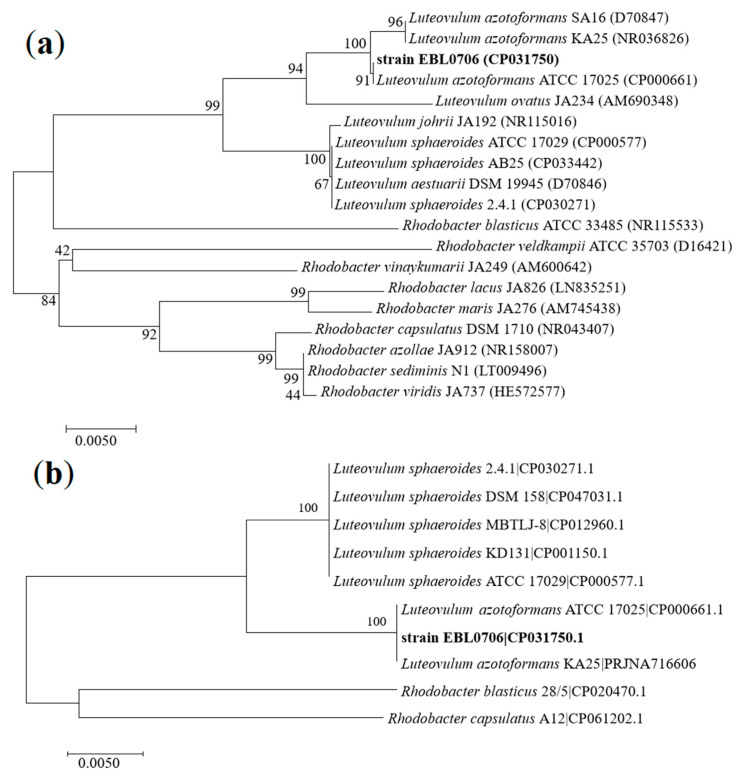
Phylogenetic tree based on 16S rRNA genes (**a**) and maximum-likelihood tree based on 20 single-copy core gene sequences showing relationships between *Luteovulum* spp. and *Rhodobacter* spp. strains; the numbers at the nodes are bootstrap values based on 1000 replicates (**b**).

We found that the 16S rRNA gene sequence of strain EBL0706 shared 100% similarity with its closest type strain *L. azotoformans* ATCC 17025 (Table S7). Two phylogenetic trees were constructed to show the same results; the strain EBL0706 was found to be a sister to *L. azotoformans*. One was based on 16S rRNA genes (Figure 3a) and another was constructed according to 20 single-copy core gene sequences of reference strains in the genera of *Luteovulum* and *Rhodobacter* (Figure 3b).

### 3.4. ANI, TETRA, and dDDH Analyses

ANI, TETRA, and dDDH values between strain EBL0706 and different *Luteovulum* strains were calculated (Table 2). The ANI value of strain EBL0706 against *L. azotoformans* ATCC 17025 and *L. azotoformans* KA25^T^ were 98.13% and 99.56%, respectively. Both were higher than the defined threshold (95%). In contrast, the ANI value of strain EBL0706 against that of *L. sphaeroides* was down to 84.7–85%, indicating strain EBL0706 was phylogenetically close to *L. azotoformans*. Therefore, strain EBL0706 should be reclassified as *L. azotoformans* rather than *L. sphaeroides*. The results of TETRA and dDDH also supported the conclusion (Table 2).

### 3.5. Comparative Genome Analyses

**Figure 4:**
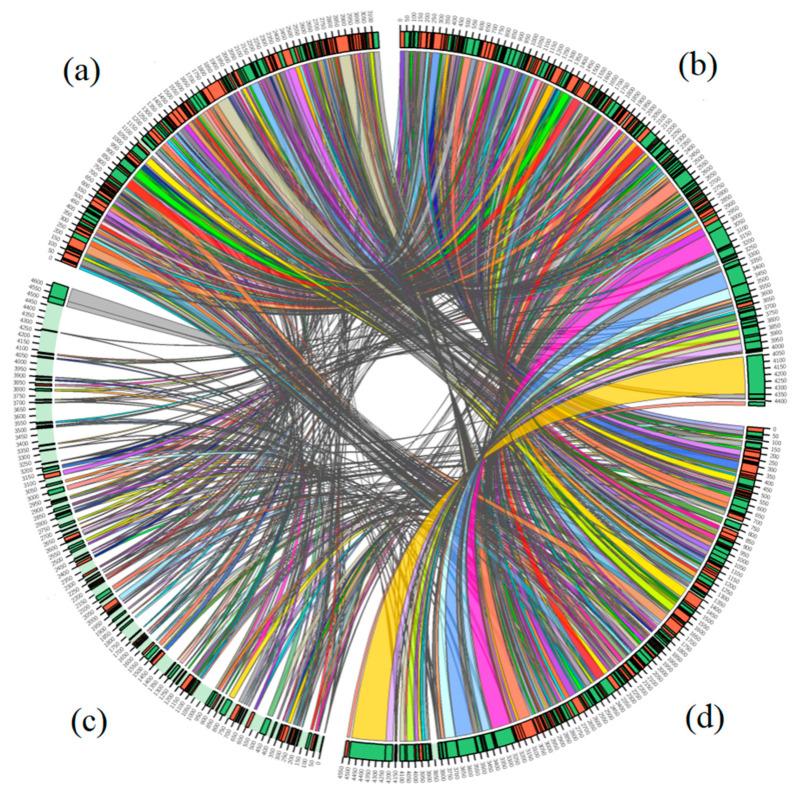
Genomic synteny shared between *L. azotoformans* KA25^T^ (**a**), strain EBL0706 (**b**), *L. sphaeroides* 2.4.1^T^ (**c**), and *L. azotoformans* ATCC 17025 (**d**).

The synteny analysis of the whole genome of strain EBL0706 and *Luteovulum* strains (*L. azotoformans* ATCC 17025, *L. azotoformans* KA25, and *L. sphaeroides* 2.4.1) were carried out. In total, 193 syntenic blocks existed among the four strains (Table S8). Strain EBL0706 has the maximum synteny with *L. azotoformans* ATCC 17025, which was 92.2% in terms of the shared region [40]. However, only 43.5% syntenic regions of EBL0706 were shared with *L. sphaeroides* 2.4.1 (Figure 4).

## 4. Conclusions

In this study, the complete genome of strain EBL0706 was analyzed. Phylogenetic investigation based on 16S rRNA genes and complete genomes revealed that strain EBL0706 was phylogenetically close to *L. azotoformans*. The ANI, TETRA, and dDDH analyses further verified the taxonomic relationship between strain EBL0706 and the species of *L. azotoformans*. The functional analysis of the whole genome sequence of strain EBL0706 indicated that this strain encoded a complete photosynthetic apparatus and shared a major part of the genomic synteny with *L. azotoformans*. Therefore, we argue that former *Rhodobacter sphaeroides* strain EBL0706 should be reclassified as a strain of *Luteovulum azotoformans*.

## Figures and Tables

**Table 1:** Genome features of strain EBL0706, *L. azotoformans* ATCC 17025, *L. azotoformans* KA25^T^, and *L. sphaeroides* 2.4.1^T^.

Genome Features	Strain EBL0706	*L. azotoformans* ATCC 17025	*L. azotoformans* KA25^T^	*L. sphaeroides* 2.4.1^T^
Genome size (bp)	4,438,291	4,557,127	4,414,500	4,629,754
G + C content (%)	68.4%	68.2%	68.4%	68.8%
Contigs	6	6	80	6
Scaffold N50 (bp)	3,014,714	3,217,726	186,783	3,188,530
Total number of CDS	4484	4503	4282	4382
tRNA	55	54	48	53
rRNA	12	12	3	9

**Table 2:** Average nucleotide identity (ANI), tetra nucleotide signatures (TETRA) and digital DNA–DNA hybridization (dDDH) analyses between strain EBL0706 and *Luteovulum* strains. The asterisk represented the result of self-comparison.

	Strain EBL0706			*L. azotoformans* ATCC 17025			*L. azotoformans* KA25^T^			*L. sphaeroides* 2.4.1^T^			*L. sphaeroides* AB25			*L. sphaeroides* ATCC 17029		
	ANI	TETRA	dDDH	ANI	TETRA	dDDH	ANI	TETRA	dDDH	ANI	TETRA	dDDH	ANI	TETRA	dDDH	ANI	TETRA	dDDH
strain EBL0706	*	*	*	98.15	0.9997	94.67	99.5	0.9999	97.96	84.93	0.9750	0.12	84.99	0.9756	0.12	85.04	0.9765	0.13
*L. azotoformans* ATCC 17025	98.13	0.9997	94.67	*	*	*	98.03	0.9997	94.63	85.06	0.9751	0.12	85.05	0.9755	0.12	85	0.9765	0.14
*L. azotoformans* KA25^T^	99.56	0.9998	97.96	98.19	0.9997	94.63	*	*	*	84.98	0.9756	0.12	85.13	0.9762	0.12	85.08	0.9771	0.13
*L. sphaeroides* 2.4.1^T^	84.84	0.9749	0.12	84.71	0.9751	0.12	84.88	0.9756	0.12	*	*	*	97.78	0.9994	92.03	97.73	0.9996	92.74
*L. sphaeroides* AB25	84.7	0.9756	0.12	84.69	0.9755	0.12	84.79	0.9762	0.12	97.49	0.9994	92.03	*	*	*	97.59	0.9998	92.28
*L. sphaeroides* ATCC 17029	85	0.9765	0.13	84.92	0.9765	0.14	84.97	0.9771	0.13	97.8	0.9996	92.74	97.87	0.9998	92.28	*	*	*

## Data Availability

The data presented in this study are openly available in the NCBI; the accession number has been listed in the article.

## Supplementary Materials

The following are available online at https://www.mdpi.com/article/10.3390/microorganisms9081754/s1, Table S1: Differential characteristics of strain EBL0706 and related species. Table S2: The list of genomes used in the study. Table S3: General feature of the strain EBL0706 genome, Table S4: List of genes associated with these GIs in the strain EBL0706, Table S5: The intact prophage identified in the strain EBL0706, Table S6: Photosynthetic gene cluster in the strain EBL0706, Table S7: Blastx analysis of 16S rRNA gene against NCBI collection, Table S8: Comparison of multiple alignment blocks between the four strains.
